# Annotations

**Published:** 1896-04-11

**Authors:** 


### Annotations.

### Professional Secrecy and Ethics in General.

The Kitson-Playfair case, and all the controversy it has aroused, brings into prominence one marked defect in medical education and professional capacity. Of all the professions the medical is brought into the most intimate contact with delicate and embarrassing situations. Yet the medical student, alone among young professional men, is never during the whole of his curriculum offered any definite instructions in the art and practice of professional business and professional conduct. We have before contended in The Hospital that chairs of ethics, or at least one general lectureship, should be established, and that attendance upon a course of ethical lectures, however limited, should be compulsory upon evey medical student before passing his final examination. In the meantime, Mr. J. J. Merriman, of Worthing, makes a suggestion to the profession as to its behaviour in legal difficulties, which is well worth bearing in mind. Says Mr. Merriman: "In medico-legal cases I have always declined to open my mouth on neither side till put into the witness-box; and as the lawyers could not know what I should then say, they have seldom troubled me to go to the court; and thus I have been enabled to attend to my own business instead of being bored with the business or legal quibbles of other people." It is an admirable rule which Mr. Merriman propounds. No doubt a good many young practitioners tell all they know before going into court, under the mistaken impression that they are obliged to answer any question which any duly employed solicitor may ask them. That is not so; and the wisest of all general rules in this matter is to do as Mr. Merriman does, and say nothing until one is duly sworn in court.

### Pensions for Asylum Officials.

No fewer than three counties are advertising for medical superintendents for their asylums. Of these posts that of the Isle of Wight is the best, and of the other two, the Kesteven is advertised at £300 and the Hereford at £400. In both cases it is stated that no pensions will be given. Now, although the pensions clause of the Lunacy Acts is a permissive one, it has been universally acted on, and we believe the non-giving of pensions is a contravention of the spirit of the Act. Be this as it may, let us see how the Kesteven and Hereford plan will work out in practice. Presumably a man will be about thirty-five years of age on appointment. The work is well known to be extremely trying and depressing, but perhaps an average man might hold office for twenty or twenty-five years, and he might save £100 a year. When he reaches the age of sixty his capital might amount to about £4,000. This, at 3 per cent., would bring him in £120 a year, and that at a time when all his powers are failing, and when little luxuries are almost essential to life. It may be said that the salary will be increased every five years or so; and so it may, but so also will his expenses. Have the Committee of Visitors of the Kesteven and Hereford Asylums then ever thought what this principle they are inaugurating will entail? What will they do with an officer, or an attendant, or a nurse when too old for duty? Will they turn them out, or will they engage deputies for them? If they do the former public opinion would be dead against them, and they would not find it easy to procure other officers or attendants in place of those dismissed. If they adopt the latter plan it will be far more expensive to the ratepayer than pensions. It is strange that this movement should proceed from Hereford and Kesteven at a time when the four Lancashire asylums, the Middlesex asylums, the Northampton Asylum, the Derby, and the Cornwall have consolidated their pensions scheme on an equitable basis. We do not know what the Medico-Psychological Association gives its members in return for their annual guineas, but if it have a voice now is the time to use it with force and purpose.

### Mr. Gilbert Bourne on the Cost of the Boat Race.

We publish in another column a letter by Mr. Gilbert Bourne, which appeared in the *Standard*, discussing The Hospital's article on "The Physiological Cost of the Boat Race." Mr. Gilbert Bourne's own admissions go very far to establish the position taken up by The Hospital, namely, that twenty minutes of such strenuous rowing as is always seen at the boat race is too much for almost any human organism to stand without some damage. Mr. Bourne admits that a "man of ordinary physique would not be able to stand the strain" at all, and that "a man of extraordinary physique would not be able to stand it if he were not carefully prepared for it." The sixteen men who rowed on Saturday week "were trained by experts for more than ten weeks before the race." This last statement, did space permit, ought to be dealt with in connection with the "physiological cost of the boat race." In our judgment, it is conclusive against Mr. Bourne's case. Our present point, however, is the actual race itself. Such a strain as Mr. Bourne himself admits, for so long a period as twenty minutes, is, we maintain, injurious. Of course, no method of physiological mensuration has ever been applied to either the Oxford or the Cambridge crew after the race—Mr. Bourne's "personal inspection" cannot be considered a conclusive scientific process—but such methods are common in physiological laboratories; and they all go to show that a severe nervous and cardiac strain, such as the boat race admittedly involves, does serious injury to the animal organism in a wonderfully short space of time. Mr. Bourne may have seen, what is by no means an uncommon circumstance, a horse ridden to death in the hunting field. In such a case it is generally the last five minutes of a hard twenty minutes or half hour's gallop that kills the noble creature. To sum up, we are not opposed to the boat race, but only to the boat race as at present carried out. Half the distance which is now rowed would give ample opportunity for showing every quality of every member of the two crews; and would make all the difference in the world to those young men upon whom the strenuous fight annually devolves. We invite, not only Mr. Gilbert Bourne, but all persons interested in what we hold to be one of the most important and characteristic features of our national life, athleticism, to consider the question raised, with intelligence and deliberateness, from the point of view we have ventured to make our own.

